# Differences in Utilization of Lower Limb Muscle Power in Squat Jump With Positive and Negative Load

**DOI:** 10.3389/fphys.2020.00573

**Published:** 2020-06-30

**Authors:** Carlos Gabriel Fàbrica, Damian Ferraro, Elia Mercado-Palomino, Alejandro Molina-Molina, Ignacio Chirosa-Rios

**Affiliations:** ^1^Department of Biophysics, Faculty of Medicine, University of the Republic, Montevideo, Uruguay; ^2^Department of Mathematics and Statistics of the Coastline, University of the Republic, Salto, Uruguay; ^3^Sport and Health University Research Institute (IMUDS), Department of Physical Education and Sports, Faculty of Sport Sciences, University of Granada, Granada, Spain; ^4^Department of Physical Education and Sports, University of Granada, Granada, Spain

**Keywords:** biomechanics, exercise, performance, vertical jump, power, lower limb, modeling

## Abstract

Jump performance is related to the ability of lower limb muscles to produce power during the push-off phase. However, it is not known if the power associated with the action of active and passive elements of the lower limb muscles change significantly in jumps with positive and negative loads. In this study, the power associated with the action of passive and active components of lower limb muscles as a whole in squat jumps (SJ) with increase and decrease in the external load is analyzed Fourteen trained male subjects (22.5 ± 2.1 years; 176.5 ± 5.4 cm; 75.8 ± 5.8 kg; BMI 24.3 ± 1.8) performed SJ on a force plate. A functional electromechanical dynamometer (FEMD) system was used to change the external load in a range of −30 to +30% of the subject’s body weight. A model comprising a mass, a spring, an active element, and a damper was used. We applied an optimization principle to determine power in center of mass (CoM) (ptot), the powers associated with active element (pact), damper (pƔ), and spring (pk) during the push-off phase. Significant differences between loading conditions for each variable were tested by repeated-measures one-way ANOVA with Bonferroni *post hoc* analysis, *p* < 0.05. Shapes of the average curves for instantaneous variation of pact, pƔ, pk, and ptot during push-off with positive loads were closer to 0% than with negative loads. As the load increased, maximum values of ptot, pƔ, and pk decreased. Only with a negative load of −30% did ptot increase significantly, this was not accompanied by changes in pact, pƔ, and pk. The load of one’s own body provides conditions for develop high pact peaks, although the maximum ptot is not achieved in that condition. The increase in negative loads produces a significant increase in ptot, but not in pact and can be interpreted as a situation in which the power delivered to the system by the action of active components is better used. The SJ with positive load, although more similar to the instantaneous changes that occur to the SJ with body weight are not gestures where high power is developed.

## Introduction

The height achieved in a vertical jump is determined by the vertical velocity of the center of mass (CoM) at the time of takeoff. Therefore, the mechanical variable determinant for vertical jump performance is the impulse (Ruddock and Winter, 2016; Winter et al., 2016). The impulse in the jump is related to the ability of lower limb muscles to produce high power during the push-off phase (Samozino et al., 2010; Ferraro and Fábrica, 2017). In other words, our muscles perform power (rate of doing work or energy transferred or converted per unit time) to generate the impulse. That muscle power can be associated with different active and passive components of the lower limb muscle tendon units (Ferraro and Fábrica, 2017).

Considering that the ability to generate high power values with muscle action is decisive in many sports (Cormie et al., 2011), the design of training programs that maximize power generation and its use is a crucial problem that coaches face (Pazin et al., 2013). The analysis of the muscular power, in particular that due to the active action of the muscles, developed during vertical jumps carried out in conditions that change movement control and organization can contribute significantly in that sense.

Two variants of a vertical jump have been most commonly employed as a multi-joint movement to assess power in the lower limbs: squat jumps (SJ) (Cuk et al., 2014; Samozino et al., 2014; García-Ramos et al., 2017) and countermovement jumps (CMJ) (Cuk et al., 2014; Loturco et al., 2015; García-Ramos et al., 2017). The CMJ technique is more similar to the movements that occur in sports situations than the SJ technique (Bosco, 2000). However, using a simple empirical model, Ferraro and Fábrica (2017) suggest that the power generated by active elements of lower limb muscles (active power) was best evaluated with SJ (Ferraro and Fábrica, 2017). Although this model is very simple, it fits the real SJ and enables researchers to make a number of specific predictions regarding the role of lower limb components. This agrees with other studies in which more complex models were used (Prokopow et al., 2005; Bobbert and Casius, 2011; Bobbert et al., 2013). The application of this simple model to different experimental situations with SJ is an interesting option for assessing active power regulation for vertical jump height maximization and for contributing to an efficient training program design.

Vertical jumps with manipulation of external loads (positive and negative) are of great interest within multiple experimental situations that could affect movement control and organization (Markovic and Jaric, 2007; Nuzzo et al., 2010; Pazin et al., 2013; Cuk et al., 2014; Jiménez-Reyes et al., 2017). Nevertheless, a particular methodological problem during vertical jump experiments with loads that have been published to date is achieving strict load control. An interesting option for load control was used by Markovic and Jaric (2007) and then by Cuk et al. (2014). In those studies, a load that mimics added or removed weight but that does not change the inertia with respect to any axis passing through the CoM was applied. However, the strategy used by those authors (with tensed rubber bands) was only able to keep the load approximately constant because during the jump, the impulse tension of the bands varies. The authors refer to that as a limitation; it is not a quantitative control of the load, and it may require large spaces for training since the length of the bands is important and the graduation of loads is limited to a game between resistance and length that makes control and progressivity difficult. On the other hand, this can imply a risk on landing, since when descending, the belts re-tension, destabilizing if it is a discharge or increasing the load on landing if it is an increase in load (downward pull). A new multiple-joint isokinetic dynamometer (Dvir and Müller, 2019), used to control the loads in different free movements (Chamorro et al., 2017; Cerda-Vega et al., 2018), would solve this problem. This system allows carrying out of a quantitative control of the load, keeping it constant during the movement variation that takes place during the impulse time and ceasing to act once the subject takes off from the ground. The SJ height tends to increase with negative loads because the output velocity will be greater. However, it is not clear what changes occur in muscle power as positive and negative charges increase. The purpose of this study is to gain a solid understanding of how and why positive and negative loading affect muscular power during jumping. We hypothesized that there are relevant changes in the muscular power components developed during push-off when comparing SJ with positive and negative loads. This hypothesis is based on the fact that by increasing or decreasing tension, the actions of some muscle groups will change due to a combination of active factors (greater active state at the beginning, for example) and passive factors (greater muscle length, for example). This can affect the different power components, altering the impulse and jump height. To address it, we used a functional electromechanical dynamometer (FEMD) for load control in a range of -30% to +30% of the subject’s body weight during the push-off phase. Then, we validated the simple empirical model with a mass, a spring, a damper, and an active element, used by Ferraro and Fábrica (2017), for all load conditions and used it to calculate the maximal power associated to each element and the total power of the system.

## Materials and Methods

### Subjects

Fourteen trained male subjects (age 22.5 ± 2.1 years; body height 176.5 ± 5.4 cm; body mass 75.8 ± 5.8 kg; BMI 24.3 ± 1.8) were selected based on their sports experience as recreational and competitive athletes in handball and soccer. The sample size for this study was based on a power analysis conducted in previous studies (Cuk et al., 2014; Feeney et al., 2016; Ferraro and Fábrica, 2017). All subjects had participated in at least 2 years of previous strength and power training and had more than 5 years of sports experience, with a minimum training frequency of three times a week and athletic proficiency. None of the subjects had any illness or injuries that would affect the test results. Before testing, all subjects were informed of the study procedures and were required to sign an informed consent. The study was conducted following the requirements stipulated in the Declaration of Helsinki. The protocol and informed consent received approval from the University of Granada ethics committee.

### Experimental Procedures

**FIGURE 1:**
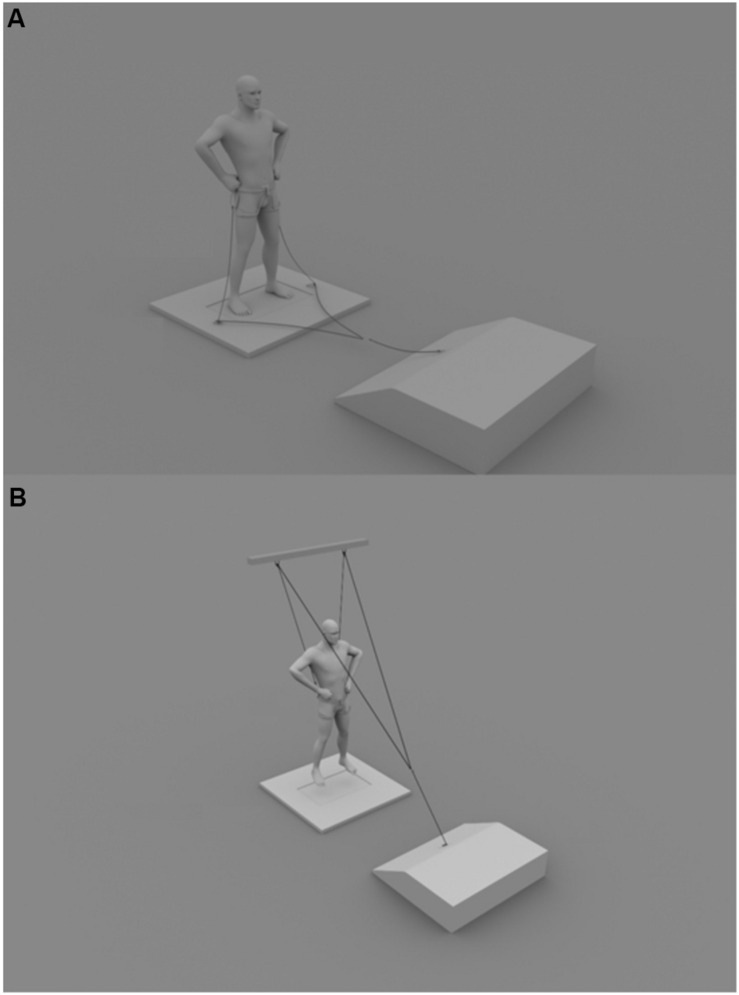
Schematic representation of the pulley system used to produce constant positive **(A)** and negative **(B)** vertical load with FEMD during SJ performance.

Subjects performed a 15-min standardized warm-up that included 5 min of continuous running, joint mobility, five skips with each leg, five heel–gluteus movements with each leg, five movements of abduction with each leg, five movements of adduction with each leg, 10 knees to the chest, and five unilateral jumps with each leg. For this study, we recorded SJ for each subject on a 50-by-60-cm piezoelectric triaxial force platform (Kistler Instruments, Hampshire, United Kingdom) varying the load condition. This platform allows data to be obtained at a frequency of 250 Hz, which could be considered low compared to other studies. However, up to 200 Hz, the ground reaction force varies less than 2% compared to a 500-Hz platform (Hori et al., 2009), so the sampling frequency at 250 Hz was acceptable. The load on the CoM was increased (10, 20, and 30% of body weight) and decreased (-10, -20, and -30% of body weight) body weight. Subjects performed five jumps in each load condition. From these, the three with the best fit to the theoretical model, those where the error term was lower (see later data processing or Ferraro and Fábrica, 2017, if a detailed explanation is required), were considered for subsequent analysis. Loads were controlled using a FEMD (Dynasystem^®^ Model Research, Symotech, Granada, Spain) in tonic mode (Chamorro et al., 2017; Cerda-Vega et al., 2018). The dynamometer pulled on each side of a climbing harness belt placed at the subject’s waist through a low-friction pulley system similar to that used by Markovic and Jaric (2007) and Cuk et al. (2014) (Figure 1). Load changes were controlled directly by the platform’s vertical component force record before the start of each jump. A design of counterbalanced measures was applied to the load order to keep an experimentally manageable number of subjects. Therefore, the 0% load condition was fixed at the beginning or end of each jump sequence. In this way, we obtained 12 combinations of load sequences and we randomly selected two for repetition. Before each jump, the participants were weighed for approximately 4 s with the external load in an upright standing position. Then, they squatted to a 90° knee flexion. After maintaining the initial position of the SJ for 3 s, which was controlled with a manual goniometer, they were instructed to jump as high as possible without performing any countermovement. The subjects’ hands remained at their waist throughout the movement. The rest time between each jump within each condition was 2 min, and there was a 5-min break between each load condition. The fatigue was monitored with the Borg (6–20) scale.

### Data Processing

Vertical force component values were exported, and a simple empirical model with an optimization principle was used. The equations and processes developed to adjust the model to jumps are described in full detail in Ferraro and Fábrica (2017). The main aspects are also explained here. The model consisted of a mass (*m*), restricted to move vertically and placed high from the ground (*h*), and two parallel components connecting the mass to the ground. The first component was a damper, and the second one was a spring (connected to the mass) followed by an active element. It was assumed that this active element represented all the active elements of the musculoskeletal complex (lower limbs) and that it was able to adjust its length (*y*) to optimize the movement for jump height maximization. The net force *f* over the mass (at time *t*) was calculated as

(1) $$f=m\,g-k\,\left(h-y-x_{0}\right)-\gamma \,\dot{h}$$

where *h* was the height of the mass, *y* the length of the active element, *x*_0_ the natural length of the spring, *k* the elasticity constant, and *γ* the damping coefficient. To maximize the jump height, the work over the mass (*W*) must be maximized during the jump time interval [0, *T*]. *W* is the integral of the power, calculated as the force over the CoM times the velocity of the CoM, over time. If the system adopts the optimal active element (*yopt*) length function that maximizes *W*, then the functional *W* (which depends on *yop*t and *h*) is maximal (as a function of *h*). Assuming this, the Euler–Lagrange equation was used to find a relationship between *h* and *yopt*. The difference between the value of *y* and the optimal *yopt* value was named *dy* and produced a force variation of *δfact = kdy*. This active external force represented a force exerted by the active element due to factors external to the model. In terms of *δfact*, the net force was calculated as

(2) $$m\,\ddot{h}=-m\,g-k\,\left(h-x_{1}\right)+\gamma \,\dot{h}+\delta \,f\,a\,c\,t$$

where *x*_1_ was a constant such that *kx*_1_ = *kx*_0_ + *a*.

When applying the model to maximum jumps, *m* was the mass of the subject, *h* the height of the CoM, and *k*, *γ*, *x*_1_, and *δfact* (Eq. 2) were assumed to be unknown. These values were calculated based on two hypotheses: the magnitude of the external active force is minimized (in the least-squares sense) during the push-off phase of a jump, and the values are different for different jump conditions. The first hypothesis was based on the fact that, if the model was correct, the active force should be completely determined by the elements considered and so the magnitude of *δfact* should be negligible. In addition, *γ* and *k* were assumed to measure the number of elements that dissipated part of the energy created by the active elements and the number of elements used to accumulate energy, respectively. Based on these assumptions, the actual values of *k*, *γ*, and *x*_1_ were those that minimize *δfact* in the least-squares sense (see Eq. 3 below). Thus, two optimization principles were used: theoretical maximization of *W*(*y*, *h*) and computational minimization of *δfact*. Values of position, velocity, and vertical acceleration of the CoM (*h*, $\dot{h}$, and $\ddot{h}$, respectively) recorded from the jumps of experimental subjects were used in Eq. 2 to adjust *k*, *γ*, and *x*_1_ to minimize the error.

(3) $$||\delta \,f\,a\,c\,t_{2}||={\left(\frac{1}{T}\,\int_{0}^{T}{\left(m\,\ddot{h}+m\,g+k\,\left(h-x_{1}\right)-\gamma \,\dot{h}\right)}^{2}\,dt\right)}^{\frac{1}{2}}$$

Once the constants *k*, *γ*, and *x*_1_ were determined, the external active force was calculated (Eq. 2), and the length *y* was computed as *y* = *k*^–1^(2*γ*$\dot{h}$ + *δfact*). In order to test the congruity between the theoretical model and the real jumps, it was assumed that in an ideal SJ, the factor *δfact* is zero. Then, the equation for the ideal movement of the CoM was assumed to be

(4) $$m\,\ddot{h}=m\,g-k\,\left(h-x_{1}\right)+\gamma \,\dot{h}$$

The general solution of Eq. 4 was adjusted to the real height in the least-squares sense. The total power over the CoM, *ptot*, was computed as *ptot* = -*f*$\dot{h}$. Following the same sign convention, the power of the active element, the damper, and the spring was defined as *pact* = *k*(*h* - *y* - *x*_0_)$\dot{h}$, *pγ* = *γ*$\dot{h}\,\ddot{h}$, and *pk* = *k*(*h* − *y* − *x*_0_)($\dot{h}$ − $\dot{\gamma }$), respectively.

Data processing was performed using Python 2.7.

### Data Analysis

Regarding the model adequacy, ——*δfact*——_2_ values, which represent the cumulative sum of errors in each frame scaled by the length of the jump, were considered. The instantaneous values of *pact*, *pγ*, and *pk* during the push-off phase were averaged for all subjects and plotted. The maximum *pact*, *pγ*, and *pk* absolute values during the push-off phase in each jump were considered to analyze the effect of positive and negative load conditions. The mean and standard deviation (SD) of those maximum values for each condition were calculated over 14 values (one for each subject), each of which was the mean of the three jumps with the closest fit to the model. This was done with the least-squares adjustment of the height of the ideal system (represented by Eq. 4) to real records of height. Data distribution was checked using the Shapiro–Wilk normality test. The significant differences between loading conditions were tested by repeated-measures one-way ANOVA with Bonferroni *post hoc* analysis. Alpha level was set at *p* < 0.05. All statistical analyses were conducted using Stata 15 (StataCorp LLC, College Station, TX, United States).

## Results

**FIGURE 2:**
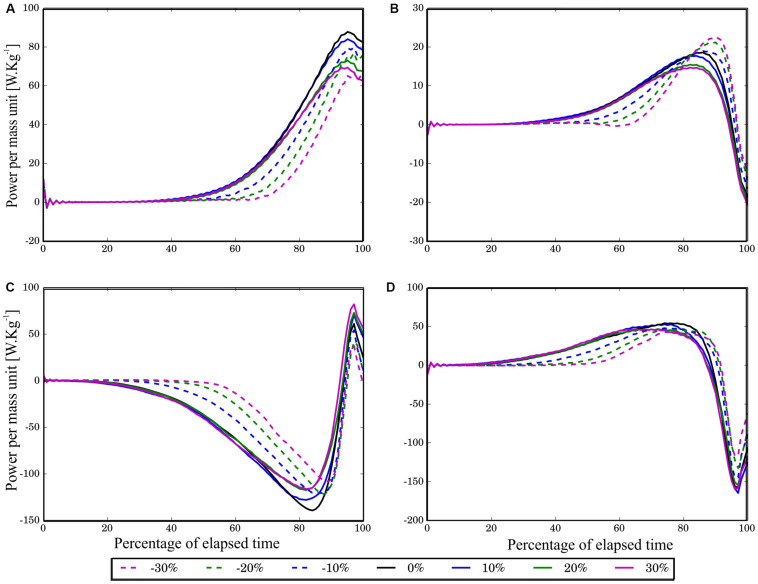
Time histories of average powers per mass unit [W⋅kg^−1^] plotted over the push-off interval expressed in elapsed time percentage. The average was calculated using the three jumps with a closer fit to the theoretical model. In order to average a group of 42 power curves (14 subjects times 3 jumps per subject), the resample function of the scipy.signal module of Python 2.7 was used to obtain signals with 100 samples. The subfigures correspond to **(A)** pγ, **(B)** *pact*, **(C)** *ptot*, and **(D)** *pk*. The continuous lines correspond to the three added load conditions and the dotted lines to the unloading conditions. The change percentage is indicated with different colors. A positive power value indicates the respective element consumes energy.

In all the jumps used for the analysis, the *R*^2^, the least squares adjustment of the height of the ideal system to real records, was greater than 0.95. The error that reflects the model adjustment for the SJ was always below 100 N for all load conditions, which represent a maximum of 5% of the average peak force reached during push-off. Figure 2 shows the average of all subjects for instantaneous *ptot*, *pact*, *pγ*, and *pk* during the normalized push-off interval for all load conditions considered in this study.

Average values (mean ± SD) of the maximum absolute values of the *ptot*, *pact*, *pγ*, and *pk* per mass unit for each load condition are presented in Table1. Note that the peak values in this table are close to those seen in Figure 2 although they do not match exactly. This is due to the way the values of Table 1 were calculated and how the graphs were constructd (see section “Materials and Methods”). In addition, note that in Figure 1, the maximal *pact* value is negative because it is power delivered to the system, but in Table 1, it is positive because the absolute value was considered for analysis.

**TABLE 1:** Average values (Mean ± s.d.) of the maximum absolute power value per mass unit for each load condition.

**Loadcondition[%]**	***pact* (Mean ± s.d) [W⋅kg^–1^]**	***pγ* (Mean ± s.d) [W⋅kg^–1^]**	***pk* (Mean ± s.d) [W⋅kg^–1^]**	***ptot* (Mean ± s.d) [W⋅kg^–1^]**
−30	117.5 (39.5)	65.9 (26.7)	51.9 (17.1)	23.9 (4.9)
–20	133.9 (46.0)	76.5 (30.7)	56.9 (19.7)	23.1 (6.2)
–10	139.8 (41.9)	80.3 (26.6)	58.8 (19.0)	20.8 (4.3)
0	152.3 (31.6)	88.9 (19.7)	66.9 (15.9)	20.6 (3.8)
10	145.3 (30.9)	85.3 (19.3)	63.1 (14.2)	19.6 (4.3)
20	128.6 (29.9)	74.2 (17.0)	56.1 (13.6)	16.5 (3.9)
30	125.3 (30.7)	70.5 (19.3)	54.7 (12.8)	15.6 (4.1)

*The mean and s.d, for each condition was calculated with 14 values (one for each subject), each of which was the mean of the three jumps with a closer fit to the model.*

Table 2 shows the Bonferroni *post hoc* analysis considering the changes within each condition. Alpha level was set at *p* < 0.05.

**TABLE 2:** Bonferroni *post hoc* analysis considering the changes within each condition.

**Load condition compared**	***pact***	***p*γ**	***pk***	***ptot***
0 % vs 10%	1.0	1.0	1.0	1.0
0% vs 20%	0.015	0.016	0.007	<0.001
0% vs 30%	0.004	0.002	0.002	<0.001
10% vs 20%	0.168	0.121	0.182	0.001
10% vs 30%	0.056	0.015	0.060	<0.001
20% vs 30%	1.0	1.0	1.0	1.0
0 % vs –10%	1.0	0.424	1.0	1.0
0% vs –20%	0.790	0.171	0.830	0.274
0% vs –30%	0.036	0.009	0.048	0.049
–10% vs –20%	1.0	1.0	1.0	0.386
–10% vs –30%	0.417	0.785	0.533	0.085
–20% vs –30%	1.0	1.0	1.0	1.0

*Alphalevelwas set at p < 0.05.*

## Discussion

Numerous investigators have studied the effects of unloading and loading on power in jumping (Dugan et al., 2004; Cormie et al., 2007; Markovic and Jaric, 2007; Nuzzo et al., 2010; Vuk et al., 2012; Pazin et al., 2013). However, this is the first study where strict control of the load on the push-off is carried out and where the action of passive and active components is considered separately. In the present study, we established the hypothesis that relevant changes occur in the generation of active power and energy dissipation associated with the joint action of the passive elements of the lower limb muscles during the push-off of SJ carried out with positive and negative loads. To address it, we used an FEMD for the first time in a study with jumps, which enabled us to keep the load constant during the push-off phase, whose control was previously monitored with the force platform. Then, we used a simple empirical model, with a mass, a spring, a damper, and an active element, to analyze the power related to each element during positive and negative load changes.

Although the selected model is extremely simple compared to many of those presented in the literature for the analysis of vertical jumps (Prokopow et al., 2005; Bobbert and Casius, 2011; Bobbert et al., 2013; Bobbert, 2014), it enabled us to make a number of specific predictions regarding the role of different components of the lower limbs (Ferraro and Fábrica, 2017).

Following the criterion considered in Ferraro and Fábrica (2017), the values of error ——*δfact*——_2_ calculated in this paper enabled us to assume that the model was an adequate representation of the human musculoskeletal system’s general behavior during SJ in the load conditions studied.

The average curves for the instantaneous power variation per mass unit, *ptot*, *pact*, *pγ*, and *pk*, during the push-off phase are presented in Figure 2. These curves show that for each power type, there was a similar variation in all load conditions. However, it can be inferred that the *pact*, *pγ*, *pk*, and *ptot* curve shapes have less variation with respect to 0% load with increasing positive load than with increasing negative loads (the positive load curves are superimposed on each other and on the 0% load condition). Regarding temporal power development, an action similar to that carried out in an SJ with 0% load is maintained during the load increase but does not happen during load reduction. This is the first factor to consider in future analysis because it indicates that the actions of both active and passive muscle components as a whole with negative loads behave differently from those in jumps without load and that discharges are used to train power or to improve jumping ability.

Regarding maximum power values of the system, in previous studies where the power output (product of vertical ground reaction force and vertical velocity of CoM) was analyzed, which would correspond to our *ptot*, both the mean values and the peak values were used (Markovic and Jaric, 2007; Pazin et al., 2013; Bobbert, 2014).

As our research has been particularly focused on the ability of the muscular system to maximize the muscle power output, we have selected the maximum power values to make the comparisons. Furthermore, peak power output is an important factor for performance in jumping because to avoid premature takeoff and therewith premature termination of work production, power output must continue to increase during the push-off (Bobbert and van Soest, 2001).

As can be seen in Table 1, maximum *ptot* tends to decrease as the load increases and to increase with negative loads. This is because, during unloading situations, a constant force against gravity was applied, increasing the velocity of CoM during push-off and consequently increasing the output power.

Although *ptot* was obtained with the simulation and not calculated directly with real force and velocity data as in other papers (Markovic and Jaric, 2007; Vuk et al., 2012; Pazin et al., 2013), given the adequate fit of the model to the real data (*R*^2^ > 0.95), both powers can be considered for comparisons. Assuming this correspondence, our results do not completely agree with previous studies. For example, Markovic and Jaric (2007) found differences for the power peak between the condition without load and positive load conditions only. The *ptot* decrease found in our work for +30% condition is similar to that reported by Markovic and Jaric (2007) with elastic force equal to 30% of body weight pulling downward on the trunk. However, in our study, the *post hoc* analysis revealed significant differences for *ptot* for the load pairs 0 vs 20, 0 vs 30, 10 vs 20, and 0 vs 30% and also between 0 and -30%. About negative loads, we observe an increase while other authors indicate that an extra upward force of 30% could cause a drop of more than 10% in peak power compared with the reference condition (Pazin et al., 2013). The changes that we observe in *ptot* with positive and negative loads are closer to those reported by Bobbert (2014), although our values are higher and the differences between the same ranges of change are greater.

In short, *ptot* increases significantly when an SJ is performed with a high percentage of discharge due to an increase in the take-off velocity, and it decreases with a lower change in percentages as the load increases. These findings do not match the “maximum dynamic output hypothesis,” which states that “the optimal load to maximize the power during the jump is the body itself” (Jaric and Markovic, 2009).

The most novel aspect of our study was the analysis of the powers associated with the system components. For load increases, significant differences were observed between the same pairs as for *ptot*, except for the comparison between 10 and 20%. Therefore, we can say that the maximum values of *pact*, *pγ*, and *pk* obtained as the load increased indicate that the decrease in *ptot* is associated with a decrease in the power of each component. On the other hand, arguably, the most important result of this study is that, during negative loads, the increase of *ptot* is not accompanied by an increase of *pact*, *pγ*, and *pk*. As can be observed in Tables 1, 2, significant changes occurred both in the powers associated with the system elements and in *ptot* only when the load was reduced by -30%, but in the opposite direction to changes observed by increasing positive loads. If we consider the ideas previously discussed in Ferraro and Fábrica (2017) regarding the power associated with the model components in SJ and CMJ, we can say that with -30% load, the mechanical behavior of SJ is at its best (development of high *ptot* values) but it also results in an efficient jump since that power is developed with lower values of *pact*. The latter is very interesting since *pact* ultimately represents the action of the muscle fibers that will respond to training; in that sense, the load of one’s own body could provide conditions for active elements to develop high power peaks, although the maximum *ptot* is not achieved in that condition.

## Conclusion

Our hypothesis is fulfilled in light of the muscle power developed: what happens in an SJ with a positive load is different from what happens in a negative load. The load of one’s own body provides conditions for active elements to develop high power peaks, although the maximum total power is not achieved in that condition. The fact that maximum active power is reached with body weight indicates that this is the best condition for training it. The increase in negative loads produces a significant increase in *ptot*, but this increase is not accompanied by the increase in *pact* and can be interpreted as a situation in which the power delivered to the system by the action of active components is better used. Therefore, if someone trains with unloading, it is actually training speed, and also the development of power over time seems to change with respect to what happens with the weight itself; thus, the technique changes. The increase with positive load in SJ has similar instantaneous changes to those with body weight, but it is not a motor task where high power is developed. However, the power values are low due to the lower speed, so it could be understood as strength training. In conclusion, our results suggest that the weight load is used to train power.

## Data Availability Statement

The datasets generated for this study are available on request to the corresponding author.

## Ethics Statement

The studies involving human participants were reviewed and approved by the University of Granada ethics committee. The patients/participants provided their written informed consent to participate in this study.

## Author Contributions

CF, EM-P, AM-M, and IC-R conceived and designed the experiments and performed the experiments. CF and DF analyzed the data. CF, DF, EM-P, and AM-M interpreted the results of research. CF and DF drafted the manuscript and prepared tables/figures. CF, DF, EM-P, AM-M, and IC-R edited, critically revised the manuscript, and approved the final version of the manuscript.

## Conflict of Interest

The authors declare that the research was conducted in the absence of any commercial or financial relationships that could be construed as a potential conflict of interest.
